# Bicuspid aortic valve repair: outcomes after 17 years of experience

**DOI:** 10.1093/ejcts/ezab176

**Published:** 2021-04-23

**Authors:** Marek J Jasinski, Kinga Kosiorowska, Radoslaw Gocol, Jakub Jasinski, Rafal Nowicki, Grzegorz Bielicki, Mikolaj Berezowski, Roman Przybylski, Marta Obremska, Marceli Lukaszewski, Anna Larysz, Andrzej Kansy, Marek A Deja

**Affiliations:** 1 Department of Cardiac Surgery, Wroclaw Medical University, Wroclaw, Poland; 2 Department of Pediatric Cardiothoracic Surgery, Children’s Memorial Pediatric Health Institute, Warsaw, Poland; 3 Department of Cardiac Surgery, Medical University of Silesia, Katowice, Poland; 4 Department of Heart Diseases, Wroclaw Medical University, Wroclaw, Poland; 5 Department of Preclinical Research, Wroclaw Medical University, Wroclaw, Poland; 6 Department of Anaesthesiology and Intensive Therapy, Wroclaw Medical University, Wroclaw, Poland

**Keywords:** Aortic valve repair, Bicuspid aortic valve, Repair failure risk factors

## Abstract

**OBJECTIVES:**

This study presents the results of 17 years of experience with bicuspid aortic valve (BAV) repair and the analysis of factors associated with repair failure and early echocardiographic outcome.

**METHODS:**

Between 2003 and 2020, a total of 206 patients [mean age: 44.5 ± 15.2 years; 152 males (74%)] with BAV insufficiency with or without aortic dilatation underwent elective aortic valve repair performed by a single surgeon with a mean follow-up of 5 ± 3.5 years. The transthoracic echocardiography examinations were reported.

**RESULTS:**

There were no deaths during the hospital stay, and all but 1 patient survived the follow-up period (99.5%). Overall, 10 patients (5%) developed severe insufficiency and 2 (1%) developed aortic dilatation requiring reoperation. Freedom from reoperation at 7 years reached 91.8%. Type 2 BAV configuration [hazard ratio (HR) 3.9; 95% confidence interval (CI): 1.01–60; *P* = 0.049], no sinotubular junction remodelling (HR 7; 95% CI: 1.7–23; *P* = 0.005), no circumferential annuloplasty (HR 3.9; 95% CI: 1.01–64; *P* = 0.047) and leaflet resection (HR 5.7; 95% CI 1.2–13. *P* = 0.017) have been identified as a risk factor of redo operation. Parameters of the postoperative left ventricle reverse remodelling improved significantly early after the operation and later at 2 years evaluation.

**CONCLUSIONS:**

The repair of BAV offers good short- and mid-term results providing a significant reverse left ventricular remodelling. Type 0 BAV preoperative configuration, circumferential annuloplasty and sinotubular junction remodelling are associated with better repair durability.

**Figure:**
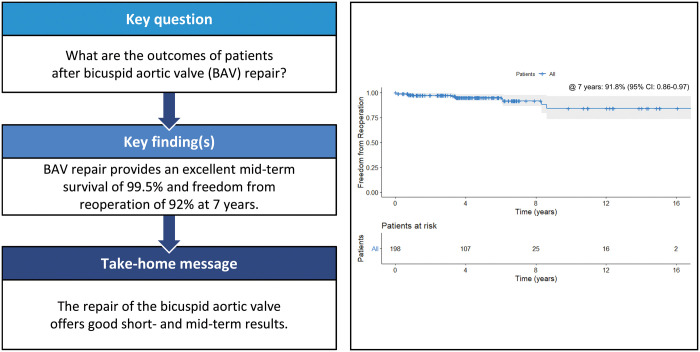

## INTRODUCTION

A bicuspid aortic valve (BAV) is the most common congenital heart defect, affecting nearly 2% of the general population [1]. The incidence of significant BAV insufficiency is much higher than that of the tricuspid aortic valve and is often associated with aortopathy. In some individuals, BAV can be successfully repaired, yet it requires a meticulous patient selection and adequate surgical experience [2]. Various repair techniques have been described so far [3–6], but individual selection is usually based on the classification of aortic regurgitation (AR) [7]. The majority of BAV repairs will remain stable over many years of observation, and survival after BAV repair is reported to be excellent [8].

This study aimed to report the mid-term clinical outcome of BAV repair, providing an analysis of risk factors for a redo and present left ventricle remodelling early after the repair and after 2 years.

## MATERIALS AND METHODS

### Ethical statement

An opinion of the local Ethics Committee was requested. The Committee decided that a follow-up was not a medical experiment, and the Committee’s consent was not required (Decision No. KNW/0022/KB/284/17, 12 December 2017; KB-558/2018, 2 October 2018). This study was approved by the local Ethics Committees of the Silesian Medical University—KNW1/146/P/10 and Wroclaw Medical University—ST.6050.18027—for statutory grants.

### Patient population

Between January 2003 and January 2020, a total of 206 consecutive patients presenting with BAV pathology were operated on by a single surgeon (Marek J. Jasinski) in the same surgical setting in 2 surgical centres in 2003–2015 at the Medical University of Silesia, and in the years 2016–2020 at the Wroclaw Medical University.

### Study design

Preoperative characteristics, procedure details, postoperative mortality and morbidity, the need for reoperation and long-term mortality were assessed. The data from transthoracic echocardiogram (TTE) examinations performed before, early after and 2 years following the surgery, according to AVIATOR (Aortic Valve Insufficiency and ascending aorta Aneurysm InternATiOnal Registry) and were retrieved from the database [7]. The minimum echocardiography follow-up time was set at 6 months.

The clinical characteristics, perioperative and follow-up data were collected directly from the patient medical records. The list of potential risk factors included baseline variables, preoperative echocardiographic data, perioperative findings and established techniques. The follow-up duration was calculated: from the operation date to the date of reoperation (the event occurred) or until 31 January 2020, the date confirmed by the Polish National Health Service that the patient is alive (event-free patients). Follow-up data on the mortality and freedom from reoperation status were ascertained based on patients’ visits in an outpatient clinic, telephone contact with the patient or patient’s relatives or the Polish National Registry of Cardiac Surgery Procedures (KROK). The above data were enhanced with data on mortality from the National Health Fund (a national public insurer). TTE findings were routinely evaluated for repair durability and to detect any significant AR ∼2 years after the procedure. According to the study design, the clinical follow-up was fully completed at the end of January 2020, simultaneously with the last KROK update.

### Echocardiographic analysis

The echocardiographic analysis included examinations performed before and early after the surgery and at 2 years (mean: 31 ± 24.3 months). The early period of TTE evaluation was defined as ranging from 1 to 6 months after the surgery. Assessed parameters included the presence and level of AR, mean and peak transaortic gradients, left ventricle end-systolic and end-diastolic dimension and volume and the dimensions of the aortic annulus, aortic root, sinotubular junction (STJ) and ascending aorta.

Mild regurgitation (grade 1) was reported if the vena contracta was <3 mm, and the central jet width was below 25% of the left ventricle outflow tract, and there was a normal flow pattern in the descending aorta. Moderate residual regurgitation (grade 2) was defined by a vena contracta of 3–6 mm and a jet width between 25% and 65% of left ventricle outflow tract. These could have been accompanied by some degree of diastolic flow reversal in the descending aorta but with end-diastolic velocity lower than 20 cm/s. Higher values of these parameters indicated moderately severe (grade 3) or severe (grade 4) with vena contracta above 6 mm.

### Procedural details

The preoperative TTE findings included the severity and character of AR, the occurrence and the location of leaflet prolapse, detailed bicuspid anatomy and the pliability or calcification of the cusps. All the operations followed a similar protocol described elsewhere (Video 1) [9]. Briefly, in all cases, the chest was opened via median sternotomy. A standard-setting cardiopulmonary bypass was initiated, and the myocardium was protected with either blood or del Nido cardioplegia. First, alignment of leaflets, the effective height of each leaflet [9, 10] and the central leaflet coaptation and individual leaflet prolapse were evaluated. Secondly, the relative lengths of the leaflet free margins were assessed [11] by suturing 2 noduli Arantii together and identifying leaflets with excessively stretched, elongated segments producing prolapse. Then, the fused leaflet anatomy was evaluated. In the case of concomitant bicuspid aortopathy, either aortic root reimplantation or STJ remodelling with supracoronary ascending aortic replacement for ascending aortic diameter ≥45 mm were performed. Sizing of the aortic graft and the external ring was based on the height of leaflets and the height of the subcommissural triangle between the left and non-coronary sinuses and the non-coronary leaflet, according to the El-Khoury and David-Feindel formulas [11, 12]. The commissures were located at 160–180° angles during root remodelling or valve reimplantation to maintain the symmetry of the repaired BAV and to enhance the fused leaflet mobility.

The enlarged aortic annulus was stabilized using either subcommissural plication or annuloplasty (SCA) or circumferential annuloplasty techniques: the internal ring annuloplasty or external annuloplasty (EA). SCA was performed with 2 braided 2–0 pledgeted stitches to narrow 2 subcommissural triangles. Internal ring annuloplasty and gore-tex suture implantation have been presented previously [11, 13]. The EA procedure consisted of the placement of a circular line of 6–8 interrupted pledgeted 2–0 braided sutures from inside of the aorta, with another 5 along the fibrotic part of the annulus and additional ones at the bottom of the third subcommissural triangle and leaflet nadir, supported by a Dacron ring placed from the outside. In other circumstances, EA was part of the reimplantation procedure [5]. The STJ remodelling was part of the supracoronary Dacron graft implantation. The sizing of the Dacron graft in both cases was identical. All patients underwent various aortic annulus and aortic root procedures, including ones without any type of annuloplasty with or without root remodelling, or with SCA with or without STJ remodelling or aortic root remodelling, and with circumferential external or internal annuloplasty with concomitant STJ and root remodelling or as an integral part of reimplantation procedure (Table 1). The leaflet repair techniques included prolapse management, raphe excision, enhanced with a patch reconstruction when tissue quality required. The Gore-Tex leaflet stabilization with Gore 7–0 or plication with monofilament 6–0 was added when necessary to correct the remaining prolapse. In all BAV repair cases, the alignment of leaflets was first, followed by the annuloplasty and the final leaflet repair. In BAV type 0 repair; both leaflets prolapse has been treated by symmetrical plication to achieve proper effective height. In BAV type 1, repair of the reference, non-fused leaflet prolapse, has been addressed first. The correct height of the reference leaflet was determined with the specially designed caliper measuring. The aim was to achieve an effective height greater than 9 mm or equal to 50% of the leaflet or root height. This leaflet repair has guided the extent of fused leaflet management. Leaflet shaving was often added to release the leaflet retraction. Root repair and its plication at the leaflet fusion level were performed, as described earlier. In BAV type 2, the linear closure of the major cleft fused leaflet was carried out, followed by the prolapse plication. A commissurotomy of a minor right-non-coronary commissural fusion was performed if a unicuspid configuration was present. When both major commissural fusions were present resulting in elevated gradient patient were not selected for the repair.

**Table 1:** Patient demographics, clinical characteristics and surgical details

Demographics	*N* = 198
Age (years)	43 (35; 56)
Males, *n* (%)	150 (73)
BMI	27 (24; 30)
ASI	2.1 (1.8; 2.5)
EuroSCORE II	2.2 (1.2; 3,7)
NYHA I	85 (41)
NYHA II	107 (52)
NYHA III	16 (8)
Comorbidities, *n* (%)	
Hypertension	113 (55)
COPD	6 (3)
Diabetes mellitus	6 (3)
Chronic renal failure	10 (5)
Surgical techniques	*N* = 198
Leaflet plication	145 (70.4)
Resection	70 (34)
Pericardial patch	8 (4)
Gore-tex	19 (9.2)
Shaving	77 (38.8)
External circular annuloplasty (Dacron ring)	92 (45)
Internal circular annuloplasty	4 (2)
Gore-Tex reinforcement	31 (15)
Subcommissural annuloplasty	81 (40)
Concomitant procedures	
STJ remodelling + aorta	125 (58)
Root remodelling/repair	49 (25)
Reimplantation—overall	21 (10.5)
Reimplantation—David V-Valsalva graft	12 (6)
Reimplantation—David I procedure	9 (4.4)

Categorical variables are given as counts and percentages and continuous variables are given as a median (first quartile; third quartile).

AR: aortic regurgitation; BAV: bicuspid aortic valve; COPD: chronic obstructive pulmonary disease; NYHA: New York Heart Association; STJ: sinotubular junction.

A transoesophageal echocardiogram was routinely performed after the induction of general anaesthesia, before the initiation of cardiopulmonary bypass and at the end of every surgical procedure. The coaptation height was considered acceptable if it was ≥4 mm after aortic valve repair. The postoperative transoesophageal echocardiogram criteria were based on quantitative parameters. Valve evaluation included measurement of the aortic annulus, the presence of coaptation and assessment of effective coaptation height and analysis of residual regurgitation, including quantification of regurgitation jet and its direction. The severity of AR (grades 1–4) was assessed according to the same criteria used in TTE. Transvalvular gradients were determined by the continuous wave Doppler method. Moderately severe (grade 3) or severe residual regurgitation (grade 4) required repeated aortic valve repair or conversion to aortic valve replacement.

### Statistical analysis

Categorical variables are presented as absolute values and percentages. Continuous variables are expressed as mean ± standard deviation for normal distribution or medians with interquartile ranges (IQR) for not-normal distribution. The Shapiro–Wilk test was used to assess the normality of the data distribution. The statistical analysis was carried out using descriptive statistics, the χ^2^ test for categorical data, the signed Mann–Whitney test was used for continuous variables that were not-normally distributed and the paired *t*-test for normally distributed. Overall freedom from reoperation was determined by using the Kaplan–Meier method. Given the descriptive nature of this analysis design and the limited number of adverse events, quantitive comparison by multivariable analyses were not our attempt. Instead, risk factors influencing reoperation were identified applying the univariable Cox PH regression method. Kaplan–Meier time-to-event curves were generated, and univariable log-rank tests were employed to compare outcomes relative to those groups. Data analysis was done using Statistica 7, MedCalc and R-statistical software.

## RESULTS

A total of 206 consecutive adult patients [mean age: 44.5 ± 15.2 years; 152 males (74%)] who underwent elective BAV repair with or without ascending aorta replacement and with or without aortic root reimplantation or remodelling were included in our final analysis. Of these, 8 patients underwent conversion to aortic valve replacement and were excluded from our final analysis. Finally, a total of 198 patients [median age: 42 years (IQR 23; 148 males (74%))] who underwent an elective aortic valve-sparing surgery for chronic AR with or without concomitant aortic root and ascending aorta dilatation were included in our study. Among the indications for repair, 121 patients presented with moderate-to-severe AR with significant dilatation of the aorta. Of these, 27 patients had moderate AR with severe aorta enlargement. There were 24 patients with severe aorta dilatation and mild AR. Only 1 patient had no evidence of AR but presented with severe dilatation of the aorta. Out of all patients, 52 were operated on due to severe AR with normal diameters of the aorta. Of all patients, 7 (3.5%) underwent concomitant coronary artery bypass grafting, 2 (1%) additional mitral valve procedure and 1 (0.5%) additional tricuspid valve surgery. The median cardiac ischaemic time was 77 (36) min, while the median cardiopulmonary bypass time was 96 (46) min. Clinical, demographic and detailed operative techniques data are presented in Table 1. A modified Sievers classification was used, where BAV type 0 is defined as symmetric with no raphe, BAV type 1 with 1 raphe and fused leaflet, with 1 dominant sinus, and BAV type 2 as unicuspid or intermediate type, with 3 equal sinuses presence and major cleft or incomplete leaflets fusion. In unicuspid valves an additional major fusion was present. The distribution of patient was as follows: BAV 0 (*n* = 46), BAV 1 (*n* = 134; 128 with left-right fusion and 8 with right-non-coronary fusion) and BAV 2 (*n* = 18) [14]. Clinical, demographic and surgical data are summarized in Table 1.

### 30-Day outcomes

There were no deaths in the 30-day postoperative period. Six (3%) patients required revision for bleeding, another 6 (3%) required pacemaker implantation and the other 6 (2%) were reoperated for bleeding. Six (3%) patients developed low cardiac output syndrome, including 2 requiring support with an intra-aortic balloon pump. One patient required prolonged ventilation, and another one developed acute renal failure. There was also 1 patient who exhibited deep sternal wound infection postoperatively.

### Late outcomes

**Figure 1:**
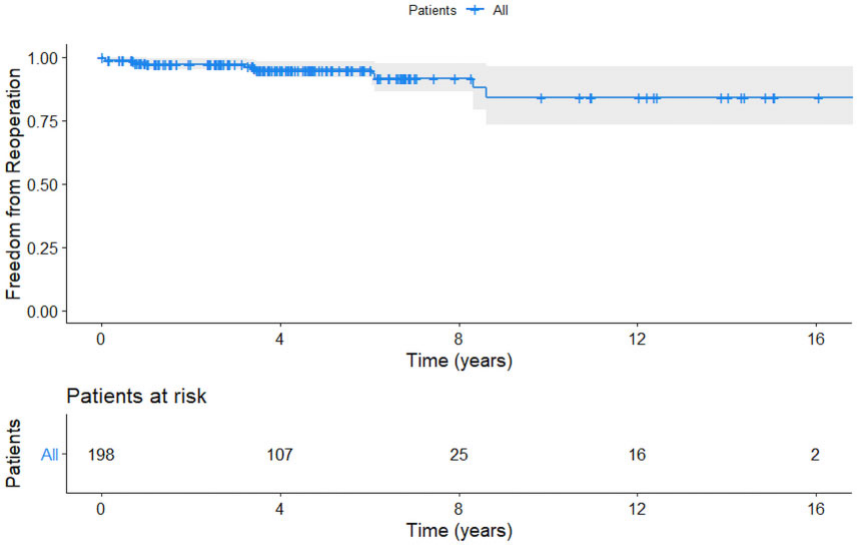
Kaplan–Meier curve demonstrating freedom from reoperation in overall study population (*N* = 198).

All patients but 1 survived the follow-up period (mean: 5 ± 3.5 years with a range of 0–17 years), consisting of 0.5% mortality rate. The cause of death was multiorgan failure developed after reoperation, which was performed 3.5 years after the primary surgery due to severe late postoperative recurrent AR. The comprehensive echocardiographic results 2 years after the surgery showed a significant positive remodelling of the left ventricle. The Kaplan–Meier estimated actuarial freedom from reoperation was 91.8% [95% confidence interval (CI): 0.86–0.97] at 7 years (Fig. 1), and freedom from structural valve deterioration was 92.3%. Fifteen patients (8%) developed structural valve deterioration of whom 13 (6.6%) developed moderate-to-severe AR, and 12 (6%) were reoperated. The primary surgery of these patients was performed: in 2006 in the case of 4 patients, in 2007 and 2010 in 2, in 2014 in 1 and in 2015 in 3. There was a significant decrease in the frequency of redo operations (*P* < 0.001) since 2012, which corresponds to the second half of the study. Reoperations were performed: due to a postoperative ventricular septal defect at the level of the perimembranous septum caused by the rupture following subcommissural annuloplasty (*n* = 1), or failure and dehiscence of raphe resection (*n* = 3), following commissural reconstruction failure (*n* = 2), due to dehiscence of patch replacement of the non-coronary cusp perforation (*n* = 1), or valve stenosis (*n* = 2) and recurrency of BAV prolapse (*n* = 1). The remaining 2 patients required aortic reoperation. The first patient returned 8 years after primary surgery with recurrent AR and progression of STJ dilatation and was successfully treated by re-repair with STJ remodelling with a Dacron graft while sparing the aortic valve. The second patient, who initially underwent BAV type 0 repair combined with partial root remodelling, developed aortic root dilatation after 3 years and thus underwent a successful Bentall de Bono root replacement.

### Risk factors analysis

**Figure 2:**
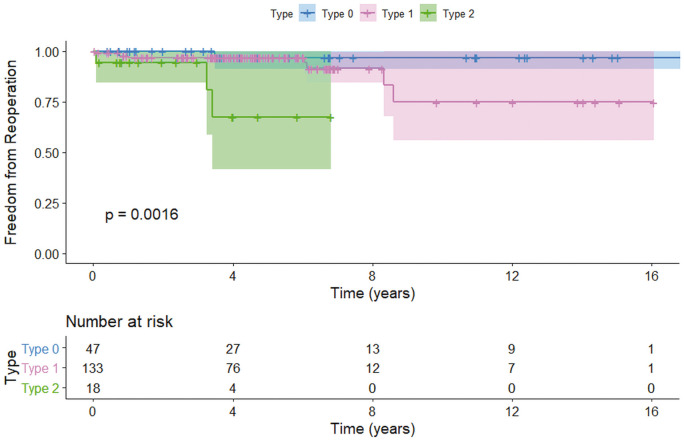
Kaplan–Meier curves demonstrating freedom from reoperation based on the modified Sievers bicuspid aortic valve cusps classification (*P* = 0016).

**Figure 3:**
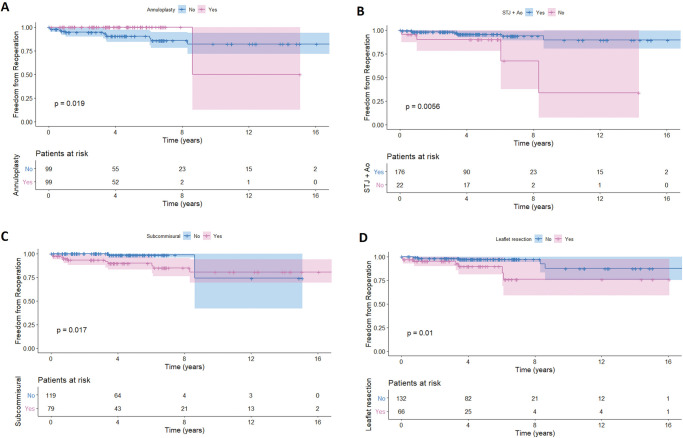
Kaplan–Meier curves demonstrating freedom from reoperation in patients whether or not (**A**) circumferential annuloplasty (*P* = 0.019), (**B**) sinotubular junction remodelling (*P* = 0.0056), (**C**) subcommissural annuloplasty (*P* = 0.017) or (**D**) leaflet resection (*P* = 0.01) was performed.

**Figure 4:**
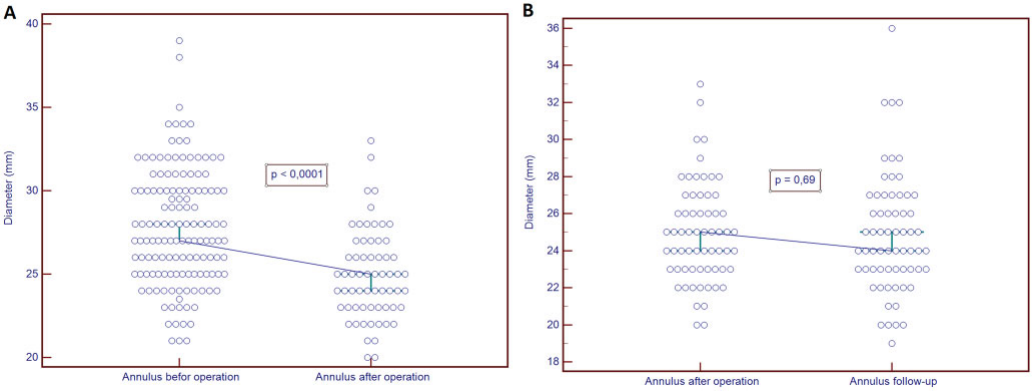
Graphs presenting the change in diameter of the aortic annulus (**A**) before and after the operation (*P* < 0.0001) and (**B**) after the operation and at follow-up (*P* = 0.69).

**Figure 5:**
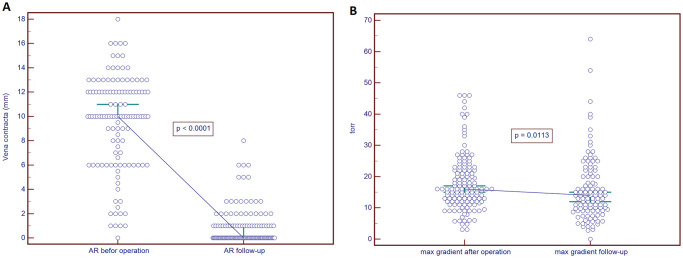
Graphs presenting the change in vena contracta (**A**) before and at follow-up (*P* < 0.0001). The second graph (**B**) presents the distribution of the peak aortic gradient after the operation and at follow-up (*P* = 0.0113).

Cumulative freedom from reoperation was calculated and illustrated with the Kaplan–Meier method. The Kaplan–Meier curves illustrate the differences between risk groups. The statistical significance of parameters was calculated with a log-rank test (Figs. 1–5). Using univariable Cox PH regression method and log-rank statistics, we noted that during mean follow-up of 5 ± 3.6 years the statistically significant risk factors associated with an increased risk of reoperation included asymmetrical BAV type 2 versus symmetrical BAV type 0 configuration [log-rank *P* = 0.0016; hazard ratio (HR) 16.8; 95% CI: 4.9–85; *P* < 0.001], no STJ remodelling versus STJ remodelling (log-rank *P* = 0.0056; HR 6.3; 95% CI: 1.4–16; *P* = 0.01), no annuloplasty versus annuloplasty performed (log-rank *P* = 0.0019; HR 3.9; 95% CI: 1–64.4; *P* = 0.018), presence of SCA versus no SCA (log-rank *P* = 0.0017; HR 4.6; 95% CI: 1.15–25; *P* = 0.031) and leaflet resection versus no resection (log-rank *P* = 0.01; HR 5.7; 95% CI 1.2–13; *P* = 0.017). The univariable analysis is shown in Table 2. Log-rank K-M is shown in Figs. 1–4.

**Table 2:** Predictors of reoperation—univariable model

Predictors	*P*-value	HR	95% CI of OR
Type 2 BAV	**0.0073**	26.5540	2.4160–291.8508
No circumferential annuloplasty	**0.0470**	8.1438	1.0286–64.4754
Subcommissural annuloplasty	**0.0317**	4.6136	1.16–25.6506
Leaflet resection	**0.0175**	4.0835	1.2793–13.0343
No STJ remodelling	**0.0120**	4.7528	1.4088–16.0336

BAV: bicuspid aortic valve; CI: confidence interval; HR: hazard ratio; OR: odds ratio; STJ: sinotubular junction. *P*-values below 0.05 were determined as statistically significant.

### Echocardiographic analysis

Detailed echocardiographic characteristics are presented in Table 3. Preoperative AR distribution was as follows: AR 0 (*n* = 1), AR 1 (*n* = 24), AR 2 (*n* = 27), AR 3 (*n* = 57) and AR 4 (*n* = 89). AR measured quantitatively with the regurgitant jet width was significantly reduced in both early and 2-year outcomes (Fig. 5). On the early observation the diameter of the aortic annulus was significantly reduced as was the aortic root, STJ and the ascending aorta. Annulus reduction was sustained at 2 years (Fig. 4). Throughout our observation, the parameters of the postoperative left ventricle reverse remodelling were sustained. Both end-diastolic and end-systolic volumes and gradients reduced significantly after the operation, and further at 2 years (Fig. 5). The ejection fraction gradually improved, and this was also sustained throughout the observation period.

**Table 3:** Pre- and postoperative parameters of patients (*N* = 136) assessed by transthoracic echocardiography

Echocardiographic parameters	Before surgery	Early after surgery	Follow-up (2 years)	*P*-value
Aortic annulus (mm), *n* (%)	27.0 (5.0)	25.0 (4.0)	24.0 (4.0)	**<0.001**
Aortic root (mm), *n* (%)	41.0 (6.0)	38.0 (5.0)	39.5 (5.5)	**0.002**
Sinotubular junction (mm), *n* (%)	35.0 (7.7)	30.5 (6.0)	32.0 (6.0)	**0.01**
Ascending aorta (mm), *n* (%)	44.0 (13.0)	32.0 (4.0)	32.0 (5.0)	**<0.001**
End-diastolic diameter (mm), *n* (%)	60.0 (15.0)	54.0 (11.0)^*^	51.0 (8.0)	**<0.001**
End-systolic diameter (mm), *n* (%)	40.0 (14.0)	37.0 (12.0)^*^	35.0 (9.0)	**<0.001**
End-diastolic volume (ml), *n* (%)	210.0 (130.8)	192.0 (124.0)	156.5 (57.0)	**<0.001**
End-systolic volume (ml), *n* (%)	117.0 (101.3)	135.0 (104.5)	61.0 (34.0)	**<0.001**
Ejection fraction (%), *n* (%)	58.0 (7.8)	55.0 (10.0)	60.0 (9.3)	**0.01**
Maximal gradient (mmHg), *n* (%)	18.0 (10.3)	16.0 (10.0)^*^	13.0 (10.5)	**<0.001**
Mean gradient (mmHg), *n* (%)	10.0 (7.0)	9.25 (6.0)^*^	7.5 (7.2)	**0.02**
Vena contracta (mm), *n* (%)	10.0 (5.6)	0.0 (2.0)	0.0 (1.0)	**<0.001**

* Statistical significance between early and late postoperative parameters. Values are median (interquartile range). *P*-values are calculated with the Mann–Whitney *U*-test for before surgery and 2 years after surgery. *P*-values below 0.05 were determined as statistically significant.

## DISCUSSION

The presence of BAV is associated with a high incidence of valve dysfunction, proximal aortic dilatation and an increased incidence of acute aortic events [15, 16]. Current recommendations include an earlier threshold for surgical correction and the use of valve-sparing operations in patients with bicuspid valve insufficiency [17]. However, the reported durability of BAV repair does not appear to be as good as for the tricuspid aortic valve. It may be related to connective tissue disorder, which is often the main feature of BAV [18]. The progressive annular dilatation caused by annulo-aortic ectasia may affect the repair stability, but precise data are still lacking. Therefore, our study aimed to analyse the mid-term clinical results and predictors of repair durability. In addition, we provided comprehensive echocardiographic evaluation early and 2 years after the repair.

Our study showed that circumferential annuloplasty significantly improved repair durability. At the same time, SCA was confirmed to be the predictor of valve-related reoperation. In patients without annuloplasty, there was a higher incidence of redo. This finding correlated well with a previously reported inferiority of the SCA compared to valve-sparing root reimplantation (VSRR) in patients with a large aortic annulus, where the entire aortic annulus is stabilized with pledged sutures. Vallabhajosyula *et al.* [19] found that the preoperative ventriculo-aortic junction (VAJ) diameter of 28 mm or more predicted a higher rate of recurrence of AR (grade >1+) following SCA rather than after VSRR. Interestingly, the VSRR was not beneficial in patients with the smaller annulus, and the durability of both techniques was similar. This finding underlines the influence of the aortic annulus dilation on AR. It is imperative in terms of addressing the annulus and preventing further dilation and AR recurrence.

The annuloplasty techniques are currently under clinical investigation, but it has already been confirmed that suture-based SCA may fail due to the late redilatation of the aortic root. Root stabilization with reimplantation provided better stability than SCA or no annuloplasty with aortic root remodelling alone [6]. In our study, we found a similar advantage of circumferential annuloplasty even when performed without additional root reimplantation. Other authors have recently shown promising results of internal or external rings, including suture-based annuloplasty, the Coroneo expandable ring and the HAART annuloplasty device [13, 20, 21]. The universal and reproducible technique of recreating symmetrical BAV has been recommended [13, 22]. Both Aicher *et al.* [4] and de Kerchove *et al.* [6] provided justification for 160–180° commissural orientation of the valve.

Our analysis has also confirmed the preoperative phenotype as a significant predictor for freedom from redo operation. It has recently been suggested that symmetrical preoperative commissural orientation imposes better postoperative durability [4, 23] and haemodynamics [24]. The recent study of Schäfers *et al.* presenting 15 years results of 1024 patients indicates that an asymmetrical commissural orientation is the strongest independent risk factor for reoperation what corroborates with our findings [8]. Accordingly, the newly presented classification of the 3 BAV phenotypes in terms of the commissure orientation may indicate a particular type of repair [25].

Our investigation also focused on the importance of simultaneous stabilization of STJ. As a result, STJ remodelling as a part of valve-sparing aortic replacement proved to be a significant durability predictor. The stabilization of repair is achieved not only by VAJ annuloplasty but with STJ stabilization confirmed by recent data [26]. Similar to our findings, Schneider *et al.* presented good durability of either SCA or circular VAJ annuloplasty provided that repair was simultaneously combined with the aorta replacement or root remodelling [13]. Better durability of BAV repair along with root stabilization and reimplantation was demonstrated by Boodhwani *et al.* [22].

Our data identify leaflet raphe resection to be a significant predictor of valve-related reoperation. It has been recognized that the calcification of leaflet or raphe with its resection and patch repair is strongly correlated with suboptimal durability, as recently reported by Schneider *et al.*, indicating proper preselection of aortic valve repair candidates [8].

### Limitations

First, multivariable analysis was not possible due to the low event rate. For this reason, the univariable Cox analysis was implemented to identify risk factors. Then Kaplan–Meier time-to-event curves were generated, and log-rank tests were employed to compare the outcomes relative to subgroups.

Second, CMR was not an imaging technique to assess remodelling in all patients. Instead, the echocardiographic assessment was implemented, including volumetric parameters, following applicable recommendations.

## CONCLUSION

In summary, the repair of the BAV offers good short- and mid-term results with extremely low mortality and sustained reverse LV remodelling. Symmetrical types 0 and 1 BAV were identified as a positive predictor of better repair durability. At the same time, no annuloplasty, no STJ remodelling and leaflet resection were associated with a higher risk of reoperation. We believe that the mid-term results of BAV repair may be further improved with the more aggressive annulus, root and STJ stabilization. Based on these data and the recent 20-year studies from Homburg, repairing the insufficient bicuspid valve should be the procedure of choice.

## Funding

This study was approved by the local Ethics Committees of the Silesian Medical University—KNW1/146/P//10—and Wroclaw Medical University—ST.6050.18027—for statutory grants.

**Conflict of interest:** Marek J. Jasinski is a consultant to Biostable. The other authors report no conflict of interest.

## Author contributions

**Marek J. Jasinski:** Conceptualization; Data curation; Formal analysis; Funding acquisition; Investigation; Methodology; Supervision; Validation; Writing—original draft. **Kinga Kosiorowska:** Data curation; Formal analysis; Validation; Writing—original draft; Writing—review & editing. **Radoslaw Gocol:** Formal analysis; Validation. **Jakub Jasinski:** Data curation; Formal analysis. **Rafal Nowicki:** Data curation; Validation. **Grzegorz Bielicki:** Data curation; Formal analysis; Investigation; Supervision; Validation. **Mikolaj Berezowski:** Data curation; Formal analysis; Investigation; Validation; Writing—review & editing. **Roman Przybylski:** Investigation; Supervision; Validation. **Marta Obremska:** Data curation; Formal analysis; Methodology; Validation. **Marceli Lukaszewski:** Data curation; Formal analysis; Methodology; Supervision; Validation. **Anna Larysz:** Data curation; Validation. **Andrzej Kansy:** Conceptualization; Data curation; Formal analysis; Methodology; Visualization; Writing—review & editing. **Marek A. Deja:** Supervision; Validation.

## Reviewer information

European Journal of Cardio-Thoracic Surgery thanks Masamichi Ono, John R. Pepper, J. Scott Rankin and the other, anonymous reviewer(s) for their contribution to the peer review process of this article.

## Notes

Presented at the 34th Annual Meeting of the European Association for Cardio-Thoracic Surgery, Barcelona, Spain, 8–10 October 2020.

## ABBREVIATIONS

AR: Aortic regurgitation
BAV: Bicuspid aortic valve
EA: External annuloplasty
STJ: Sinotubular junction
SCA: Subcommissural plication or annuloplasty
TTE: Transthoracic echocardiogram
VSRR: Valve-sparing root reimplantation
VAJ: Ventriculo-aortic junction
